# Working with Climate Projections to Estimate Disease Burden: Perspectives from Public Health

**DOI:** 10.3390/ijerph13080804

**Published:** 2016-08-09

**Authors:** Kathryn C. Conlon, Kristina W. Kintziger, Meredith Jagger, Lydia Stefanova, Christopher K. Uejio, Charles Konrad

**Affiliations:** 1 Climate and Health Program, Division of Environmental Hazards and Health Effects, National Center for Environmental Health, Centers for Disease Control and Prevention, Atlanta, GA 30341, USA; cuejio@fsu.edu; 2 Florida Department of Health, Tallahassee, FL 32399, USA; Kristina.Kintziger@FLHealth.gov; 3 Oregon Public Health Authority, Portland, OR 97232, USA; Meredith.A.Jagger@state.or.us; 4 Center for Ocean Atmosphere Prediction Studies, Florida State University, Tallahassee, FL 32306-2741, USA; lstefanova@fsu.edu; 5 Department of Geography, Florida State University, Tallahassee, FL 32306-2190, USA; 6 Department of Geography, University of North Carolina at Chapel Hill, Chapel Hill, NC 27599-3220, USA; konrad@unc.edu

**Keywords:** public health, climate modeling, project disease burden, attributable fraction, adaptation

## Abstract

There is interest among agencies and public health practitioners in the United States (USA) to estimate the future burden of climate-related health outcomes. Calculating disease burden projections can be especially daunting, given the complexities of climate modeling and the multiple pathways by which climate influences public health. Interdisciplinary coordination between public health practitioners and climate scientists is necessary for scientifically derived estimates. We describe a unique partnership of state and regional climate scientists and public health practitioners assembled by the Florida Building Resilience Against Climate Effects (BRACE) program. We provide a background on climate modeling and projections that has been developed specifically for public health practitioners, describe methodologies for combining climate and health data to project disease burden, and demonstrate three examples of this process used in Florida.

## 1. Introduction

The wide range of health impacts related to a changing climate is garnering attention from public health agencies in the U.S. to consider how best to plan for future impacts. Extreme weather, wildfires, tropical storms, drought, and floods are expected to increase over time in the Southeastern U.S. [1]. The distribution of infectious disease carrying vectors, such as mosquitoes and ticks, is sensitive to weather and climate [2]. The impacts are already underway in the U.S. and are likely to continue, resulting in a measurable burden on overall population health [3]. From the Chicago heatwave in 1996 that resulted in more than 690 excess deaths [4] to the deadly extreme flash flood events that occurred frequently between 2006 and 2012 [1], public health officials will continue to be faced with responding to climate-related health outcomes.

Amid these growing concerns, public health practitioners are increasingly seeking to incorporate climate projection data into disease burden projections of climate-related health outcomes. Projected disease burden estimates can aid public health practitioners in planning response and adaptation efforts in anticipation of climate change. Without knowledge of the magnitude of impacts, public health practitioners may not have all the information needed to prioritize interventions and develop response plans that protect at-risk populations. Projecting disease burden can be especially daunting, given the complexities of climate modeling and the multiple pathways by which climate influences public health. Several state and local public health agencies have been funded by the Centers for Disease Control and Prevention’s (CDC) Climate Ready States and Cities Initiative (CRSCI) to employ a capacity-building framework, Building Resilience Against Climate Effects (BRACE), that supports public health agencies to anticipate how climate change will affect population health [5]. Projecting weather and climate-related disease burdens is one step in this framework. The CDC’s Climate and Health Program provides a guidance document *Projecting Climate-Related Disease Burden*: *A Guide for Health Departments* that serves as a useful starting point for health practitioners interested in characterizing the impacts of climate change on human health. The guidance focuses on four components necessary for conducting thorough disease burden projection estimation: (1) developing a causal pathway; (2) assembling data elements; (3) projecting disease burden; and (4) performing uncertainty analysis. The disease burden projection process is guided by the principle of adaptive management, which allows for management decisions to be iteratively evaluated and modified over time with stakeholder input. Within each component are methods and considerations that, when incorporated into the disease burden projection exercise, strengthen the final projection estimate.

Like many interdisciplinary projects, subject matter experts provide a thorough understanding of data sources and data products. The process of combining climatological and public health data is no different. Climatological data, such as the climate projection data products that are necessary for projecting future disease burden, may contain nuances that require technical knowledge of how those data products can be utilized for estimating future climate-related health burden. For instance, interpreting the projections of climate models and working with the inherent uncertainties in the projections may not fall under the expertise of a public health practitioner. Just as the climatologist has expertise in climate models, epidemiologists are experts in studying how an exposure can impact human health, considering the social and behavioral factors that contribute to health. A public health practitioner is best suited to determine the appropriate epidemiological methods to identify the health impacts of a climate exposure (e.g., decreased precipitation), as well as how to estimate the present day and future health burden. Thus, coordination of technical experts is imperative for rigorous science that spans the climatological and epidemiological fields.

The Florida BRACE program spent over two years building capacity between public health practitioners and climatological experts. The partnership identified the need for a climate modeling guide geared toward public health professionals, with a specific focus on how the climate model outputs can be applied to projecting disease burden. The goal of this paper is to present an overview of the technical considerations needed from both the climatological projections and epidemiological perspectives when estimating future climate-related health burden. The first half focuses on climate, providing a primer on climate change and variability, the models used to characterize global climate, and uncertainty in the models. The second half presents the processes for selecting and applying climate projection data to estimate health burden of climate-related exposures. An interdisciplinary team assembled by the Florida BRACE program worked closely to project disease burden. We provide detailed examples from this collaboration to illustrate the technical and methodological requirements needed to project disease burden related to three distinct climate exposures: (1) drought; (2) high temperatures; and (3) tropical cyclones, and present results from one projected disease burden, heat related illness (HRI).

### 1.1. Introduction to Climate Change

A basic understanding of climate and how it varies with respect to time is crucial for any end-user of climate projection data. Of particular interest are the natural variations in climate and how they are distinguished from long-term changes in climate, especially changes associated with the increase in greenhouse gases like carbon dioxide (CO_2_) and methane (CH_4_).

#### Climate, Climate Change and Climate Variability

Climate is defined as the weather conditions that typically exist in a place or region. The climate of the Southeastern U.S., for instance, displays some degree of variability in the weather from day to day, week to week, and across the seasons. There are periods of relatively cool or warm weather that may last a day or two or extend out a week or more, especially during the winter months. While climates are typically summarized in terms of their averages, they are best described in terms of the variability and range of weather conditions that are typically experienced. For instance, Tallahassee, Florida, U.S. has a climate that is characterized as very warm and moist during the summer, with a daily mean maximum temperature of 33.1 °C (91.5 °F) and 56.5 cm (22.2 inches) of precipitation on average [6]. Describing how frequently the weather exhibits a state change on average (e.g., the number of days the temperature exceeds a certain level, or how often it rains) may be of particular interest to public health practitioners concerned with extreme events. The frequencies of extreme events are typically described in terms of return period, which is the average period of time between two extreme events of a given magnitude or greater. In Tallahassee, once every 10 years on average, the city receives 18.8 cm (7.4 inches) or more of daily rainfall [7]. This is an average measure and does not imply that weather extremes occur on a regular cycle. Several types of extreme events show clustering tendencies with respect to time. Specifically, extended periods are found in the historical record in which few, if any, extreme events are observed. These time periods are then punctuated by relatively short periods in which the extreme events occur multiple times. This clustering of extremes is tied to climate variability [8].

**Figure 1:**
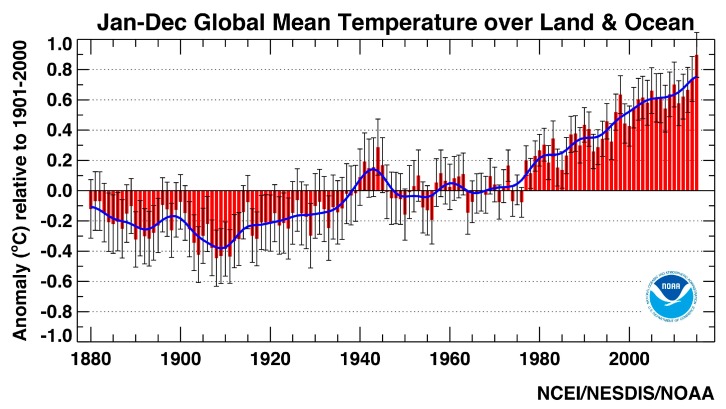
The annual trend in global temperature (°C), expressed as a departure from 1901 to 2000 average [10].

The climate of a given place or region is typically determined through an analysis of weather records that are available over extended time periods, typically 30 to 100 years or more [9]. Climate, therefore, provides a long-term perspective on the prevailing conditions or averages. Climate change identifies the changes in the climate that occur on time scales from several decades to millions of years. In order to quantify climate change, weather records are required that extend beyond the time period in which short-term natural climate variations occur. The analysis of global temperatures (Figure 1), for example, reveals alternating warm and cool periods in the record, but a long-term increase in temperature (e.g., the annual mean temperature shown as a blue line) across the 100 plus-year period of record.

Climate variability, on the other hand, identifies short-term variations or departures from this typical or average climate state (e.g., one season to several decades). Inter-annual variations in the climate can be significant with relatively warm or dry years followed by exceptionally cool or wet years (e.g., Figure 1). Likewise, inter-decadal variations in climate occur in which relatively warm or dry periods are followed by relatively cool or wet periods. Global temperatures, for example, cooled slightly between the 1950s and 1970s [11], in spite of the long-term warming trend.

Climate variability is especially pronounced across certain regions of the U.S. The northern interior portion of the U.S., for example, displays greater temperature variability than the southern U.S. and coastal regions. The western U.S. experiences much greater year-to-year variability in precipitation [12], with the frequent occurrence of extended dry conditions, vividly illustrated by recurrent drought in California [13] that are punctuated by short periods of heavy rainfall and flooding.

One major source of climate variability is fluctuations in sea surface temperature (SST) over ocean basins [14], which occupy more than two-thirds of the earth’s surface. A very good example of this is the El Niño phenomenon. El Niño is a recurring pattern of climate variability that occurs when increased SSTs are present for several consecutive months across the equatorial central Pacific. It produces numerous powerful thunderstorms that, in turn, cause changes in the general atmospheric circulation around Earth. These circulation changes produce significant short-term changes in the climate over various portions of the world. During an El Niño event, for example, portions of southern California and the southern U.S. are likely to experience above-normal amounts of precipitation [15,16,17], which often leads to flooding [18]. In addition, upper level winds with an El Niño event inhibit the development of hurricanes over the Atlantic Ocean [19]. The back-and-forth interactions between the sea surface and the atmosphere are complex and not yet fully understood. Climate models are generally accurate in representing the influence of El Niño on the atmosphere, once El Niño is present in the ocean. However, since the El Niño phenomenon itself is the result of interactions between the ocean and the atmosphere, climate models have difficulty producing accurate representations of what occurs to the entire system when El Niño is present.

### 1.2. Global Circulation Models

A global or general circulation model (GCM) utilizes physical laws and relationships to simulate large-scale circulations and processes in the atmosphere. Large-scale in this case refers to atmospheric circulations, patterns, and processes that persist or prevail across broad regions. Within these large regions, numerous smaller scale circulations and processes are found that can have a significant influence on the climate. While they are not captured by a GCM, their influence is typically modeled through the use of downscaling techniques.

GCMs estimate future changes in the state of the climate by altering one or more characteristics of the atmosphere and, in some simulations, the land surface in the model. Levels of CO_2_ and other greenhouse gases are increased by prescribed amounts according to various future emissions scenarios [20]. These scenarios may also specify long-term changes in land cover type, such as urbanization and deforestation that have subtle effects on the climate.

**Figure 2:**
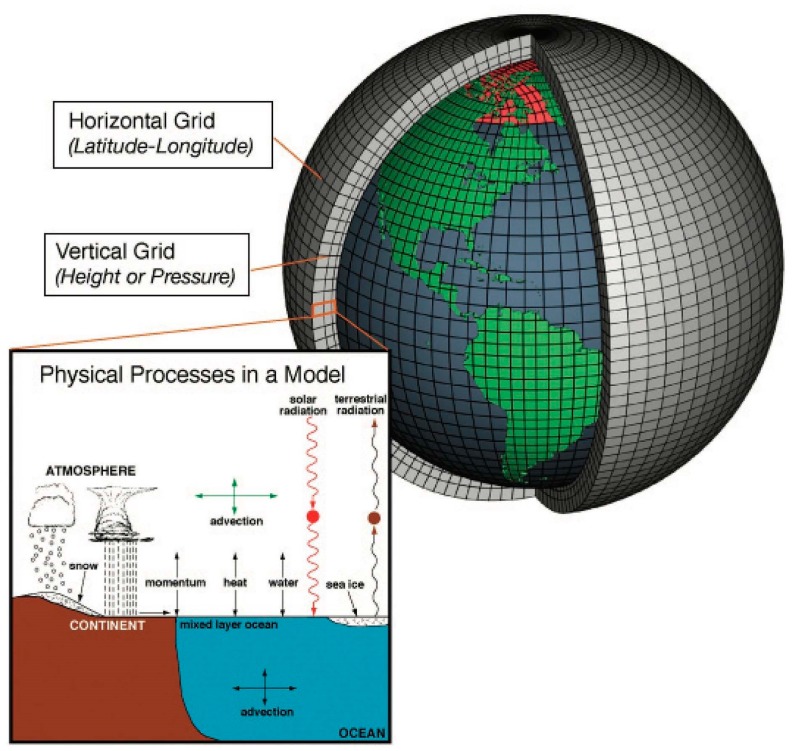
Schematic illustration of a global circulation model. From National Oceanic Atmospheric Association (NOAA) [21].

GCMs simulate atmospheric processes and circulations by dividing the atmosphere into a large number of vertically stacked boxes spaced out evenly across the earth’s surface (Figure 2). These boxes are delineated by a grid placed over the earth’s surface that is typically aligned along lines of latitude and longitude. The model carries out simulations within each box or grid space, whose length is typically between 100 and 300 km. On the bottommost box, the interacting effects of Earth’s surface and the overlying atmosphere are simulated, including the upward and downward movements of heat, momentum, and water. Over the tropical sea surface, for example, the model estimates how much heat and moisture is moved from the sea surface into the atmosphere. Because oceans cover much of the planet, the movement of water vapor between the sea surface and the atmosphere have a huge influence on the GCM simulations. Movement of heat within the oceans influences prevailing ocean currents and circulations, and, is thus accounted for in most models. Many atmospheric processes and features, such as precipitation and clouds, occur in patterns that are typically much smaller than the boxes within which the atmosphere is simulated. Because of this mismatch in scale (i.e., difference in area in which they occur), they cannot be explicitly modeled. To overcome this limitation, climate modelers develop parameterizations, which are statistical representations of the collective effects of these processes and features across each individual grid box. For example, a simple regression equation might be used to estimate the cumulative effects of convection (e.g., thunderstorms) across a given grid box using the vertical rate of temperature change as the independent variable.

Climate projections are provided in the form of gridded datasets, each describing future states of the atmosphere across regularly spaced boxes within a grid laid out across the earth’s surface (e.g., intersecting points of lines running north-south and east-west). Climate scientists typically develop downscaled projections of surface temperature and precipitation, which are expressed in the form of a daily or monthly time series. The values of daily mean temperature and daily total precipitation, for example, can be retrieved for a selected grid box in a given region for a specified period in the future (e.g., daily time series from 1 January 2040 to 31 December 2069). A climate projection is typically summarized through the calculation of various statistics, including: (a) the average of a meteorological variable across a month, season or annual period (e.g., mean monthly temperature, mean annual precipitation, etc.); and (b) the frequency with which a given threshold is equaled or exceeded {e.g., the number of days in which the daily maximum temperature equals or exceeds 35 °C (95 °F), etc.} [20].

### 1.3. Downscaling Climate Model Projections

GCMs operate at a coarse scale (e.g., 100–300 km boxes). Computational constraints, however, prevent them from simulating the myriad smaller scale processes (e.g., clouds and turbulence) and the impacts of terrain (e.g., mountains, lakes, bays, differing land cover types) that play a key role in shaping regional to local scale climates. Also, the coarse resolution of climate models prevents them from simulating changing weather conditions and short-term climate variability, both of which are tied to the occurrence of impactful climate extremes at a regional to local scale. Further, the coarse resolution of GCMs prevents simulating the development and movement of regional scale weather systems, such as hurricanes and mid-latitude cyclones. In order to capture the influences of weather systems and smaller scale surface features on the climate, GCM outputs must be downscaled. Climate scientists have connected GCMs with fine-gridded downscaling models that provide a detailed perspective on the climate at a regional to local scale.

#### 1.3.1. Dynamical Downscaling

Dynamical downscaling employs a regional climate model (RCM) that operates over a much finer grid (e.g., 5–50 km). An RCM functions very much like a weather model, iterating over time increments of several minutes to simulate changing weather patterns over a region. Because of the computational expense in running the model over such a long time period, the domain of the model must be restricted to a small region of the world (e.g., the Midwestern or Mid-Atlantic States of the U.S.). Generally, available dynamical downscaling models have resolutions of several kilometers, which enable them to effectively simulate convection and resulting precipitation. For instance, the downscaling model used for the CLAREnCE10 dataset, developed at Florida State University, is able to simulate the daily sea breeze circulation along the Gulf Coast and the thunderstorms that develop and move out along its edges [22].

There are a few shortcomings to the dynamical downscaling approach. First, it requires a tremendous amount of computing resources, even on the fastest supercomputers that are currently available. As a result, only a limited number of model “runs” can be made to project future climate changes. Second, errors and uncertainties are introduced from both the GCM, whose output goes into the RCM model, and the RCM model itself.

The errors generated in the dynamical downscaling approach are commonly reduced through a practice called “bias correction”. Bias correction is an adjustment of model values of the RCM output to reflect observed and statistical properties. In order to carry this out, the model is run for a historical period, such as the last 30 years, and the output compared with the observed climate for this period. It is worth noting that the model output should not be expected to correspond with the observed climate for any given historical year. The model bias is determined by identifying statistical differences in the atmospheric variables of interest (e.g., difference in means and standard deviations). The bias-correction procedure is essentially the generation of a ‘recipe’ for translating modeled values into real world values.

Bias correction methodologies range from relatively simple (e.g., linear correction) to more complicated ones (e.g., quantile matching). Bias correction unavoidably requires the unverifiable assumption that systematic errors are constant over time. Based on historical warm and cold periods, Teutschbein and Seibert [23] have shown that linear bias correction is adequate for temperatures, while precipitation bias correction requires a more sophisticated method, such as quantile matching. Suppose an RCM is run with the output from a GCM simulation that was performed using observed greenhouse gas concentrations for the last 30 years. The simplest (and least accurate) bias correction approach is to compare the average temperature generated from this RCM for a given grid box with the average observed temperature in the last 30 years in that same grid box. If the RCM average September temperature is 20 °C (68 °F), and the observed average September temperature is 22 °C (72 °F), then the bias-correction recipe is to add the difference (2 °C/4 °F) to every September day coming from the RCM.

In practice, a more complicated bias-correction is usually applied: the values of RCM-simulated precipitation of all September days (a total of 900 days) are arranged from the smallest to the largest. Similarly, the observed daily precipitation from the last 30 years is arranged from smallest to largest. The numbers in each position are then matched. For example, if the 35th smallest value in the RCM simulation is 10 mm, and the 35th smallest value in the observations is 15 mm, it is noted that, in the future, when the RCM produces a daily precipitation of 10 mm, it is to be interpreted as 15 mm.

Dynamical downscaling is limited in the number of GCM and emissions scenarios that can be considered in future projections. However, the use of an RCM enables climate scientists to derive dynamically downscaled projections for a wide range of climate variables. It is important to note that, unless explicitly specified, dynamically downscaled datasets are typically provided without bias correction. It is essential that a bias-correction procedure be applied prior to use of such data.

#### 1.3.2. Statistical Downscaling

Statistical downscaling consists of various statistical techniques for translating climate attributes from a large scale or big area (e.g., the output from a GCM) to a smaller region or locale of interest. In order to carry this out, data from the observed historical climate (e.g., the last 30 years) are used to identify a statistical relationship between relevant large-scale circulation features and a local or regional climate variable of interest. The statistical relationship is then applied to the climate projections output from a GCM to predict the values of the climate variable at the regional level.

For instance, a statistical relationship is derived between the GCM-predicted temperatures over a large area and observed summertime temperatures in Tallahassee, FL, U.S. The resulting regression model is then applied to the future climate projections of a GCM over the same large area to predict the temperatures for Tallahassee. It provides a transfer function that translates or downscales circulation attributes over a large region into local scale projections of temperature. Bias correction is typically built into the statistical downscaling procedure, and statistically downscaled datasets usually do not require further bias correction [24,25,26].

There are strengths and limitations to using the statistical downscaling approach. Like dynamical downscaling, statistical downscaling is subject to the inherent errors in the GCM. Additionally, statistical downscaling techniques assume that the statistical relationships identified in the observed historical record between large-scale circulation features and regional/local climate will not change in the future climate. In other words, it assumes that there is stationarity (i.e., no change) in the statistical properties of the variables, like human behavior (e.g., dramatic reduction in greenhouse gas pollution) across time. Unlike dynamical downscaling, which requires tremendous computational resources for a single run, statistical downscaling approaches are much easier to perform. As a result, they can be applied to many more GCM projections and emissions scenarios. Dynamical downscaling, though much more limited in its application, offers the advantage of physics-based projections across a wider range of atmospheric variables.

### 1.4. Uncertainty in Climate Models

In order to work appropriately with climate model projections, it is useful to have a basic understanding of the uncertainties and sources of error in climate modeling. Three different sources of uncertainty are encountered.

#### 1.4.1. Scientific Uncertainty

Scientific uncertainty, sometimes referred to as structural uncertainty, relates to our scientific understanding of the atmosphere and the skill of the climate models in simulating the complex interplay of atmospheric processes and patterns. Climate scientists lack a complete understanding of the processes and patterns that control atmospheric circulation. As a result, they cannot determine exactly how sensitive atmospheric temperatures are to increases in CO_2_ and other greenhouse gases. One challenge relates to the energy exchanges between the surface and the atmosphere, especially over the oceans. It is not clear exactly how much of the additional CO_2_ in the atmosphere is taken up by the oceans. Finally, atmospheric scientists have not fully worked out the interacting influences between small and large-scale circulations, for example, thunderstorms that bubble up on a smaller scale (5–50 km) and the wind patterns that persist over a larger scale (50–500 km).

These deficits in scientific understanding contribute to errors and uncertainties in climate modeling and the simulation of future climate change. This uncertainty is further magnified because in order to estimate smaller scale influences one must use model parameterizations, which contain additional uncertainties. While computing power has increased exponentially in recent years, it is not sufficient for explicitly modeling smaller scale processes and features (e.g., convection, turbulence, clouds, etc.) that have a significant influence on large-scale circulation patterns over time.

#### 1.4.2. Natural Uncertainty

Natural uncertainty in the climate models results from the effects of climate variability. The climate fluctuates across a range of time scales, both inter-annually (e.g., a wet year followed by a dry year) and inter-decadally (e.g., several decades with cooler than normal temperatures followed by warmer than normal temperatures). Many of these changes are driven by internal earth-atmosphere system processes, such as heat fluctuations in the oceans and the aerosols released from volcanoes. Climate models are not yet able to adequately incorporate these sorts of influences. This is especially the case for phenomena, such as El Niño, which involve complex interactions between the atmosphere and the ocean.

#### 1.4.3. Scenario Uncertainty

Scenario uncertainty arises from unknowns related to the influences of human activities going into the future, and how they will affect the concentration of greenhouse gases. Specifically, various assumptions must be made regarding the projected rates of economic growth, especially in developing countries, and how much this will increase the global carbon footprint in the atmosphere. Other demographic and social variables, such as increased population rates, green industry growth, and emerging new technologies, play into this as well.

In designing their Coupled Model Intercomparison Project (CMIP3), the Intergovernmental Panel on Climate Change (IPCC) Fourth Assessment Report identified four different emissions scenarios describing projected changes in economic growth, population change, and technology change over the next 75 plus years [27]. In the National Climate Assessment (NCA) for the U.S., two of these scenarios were applied.

The **A2 Emissions Scenario** assumes a world of self-reliant independently operating countries with increasing population and regionally oriented economic development. This is often referred to as the “business as usual” scenario, as it assumes little change in the way in which the world economy currently operates.

The **B1 Emissions Scenario** assumes rapid economic growth and that population stabilizes around 2050 and declines in the years that follow. It assumes that clean and resource-efficient technologies will be introduced. This is sometimes referred to as a conservative scenario, as greenhouse gas emissions level off in the future.

#### 1.4.4. Addressing Uncertainty

All three sources of uncertainty affect climate model projections of future climate; however, the degree to which each contributes to the uncertainty varies according to the time frame of projection. Analysis [28,29] has shown that natural uncertainty accounts for the greatest proportion of the total uncertainty during the first decade of the projection. This uncertainty is especially great in the temperature projections. Scientific uncertainty accounts for the greatest proportion of the total uncertainty by the third decade of the projection, while scenario uncertainty peaks around the ninth decade of the projection [26,27].

Uncertainty is common in all types of models (e.g., climate, economic, statistical) and should be identified where possible. In order to capture the natural and scientific uncertainty, a range of GCM simulations are typically employed and called an “ensemble”, with each simulation providing a unique scheme for modeling the atmosphere. The average of ensemble members is called the ensemble mean. By comparing ensemble member projections of future climate for a given region, one can see how much variability exists across the models for a given atmospheric variable. Table 1 presents a list of the 10 downscaled GCM models used in the Florida cases studies.

**Figure 3:**
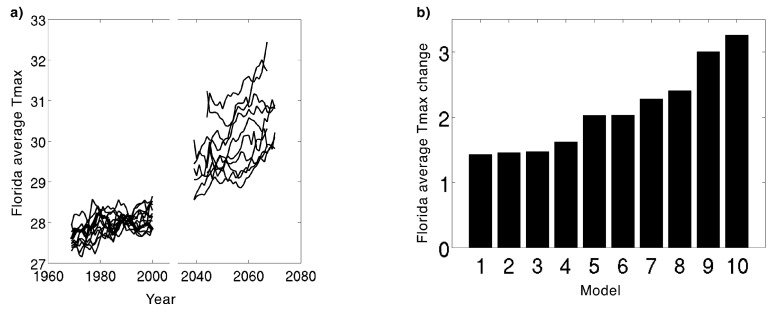
Comparison of historical simulations and future projections from the 10 downscaled models used in the Florida case studies (see Table 1): (**a**) 5-year running mean of Florida’s average annual maximum surface air temperature (T_max_ in °C) from observations for 1969–2000 (thick black line) and from the 10 downscaled model simulations for the historical (1969–2000) and future (2039–2070) projection periods; (**b**) projected change, arranged from least to greatest, in Florida’s average annual T_max_ (°C) from (1969–2000) to (2039–2070).

The difference in maximum surface air temperature projections varies across the 10 models (Figure 3). The spread or variation in the projected temperatures across the different models increases with time. This can be attributed to increased contribution of the scientific uncertainty several decades into the projection.

Climate change is projected to have differential regional effects, which translate to uncertainties in the projections that are region-specific. Uncertainties in precipitation projections, for instance, are generally greater in the southern U.S. This is due in part to the fact that a greater proportion of the precipitation results from small-scale processes and features, such as convection and thunderstorms that are not as well modeled. Uncertainties in the temperature projections, on the other hand, are relatively lower in this region of the country.

## 2. Working with Climate Projections in Public Health

### 2.1. Selecting Climate Projections

Given the uncertainties associated with climate modeling, climate projections should be viewed more as scenarios of what may happen in the future as opposed to forecasts, such as, for example, the weather forecasts for the next several days. Specifically, the wide spread in the future projections for a given climate variable (e.g., maximum temperature in Figure 3) reveals a range of possibilities in terms of what may happen. These differences result from the fact that GCMs and downscaled RCMs vary from one another in their physics packages and the ways in which they parameterize influences of smaller scale processes.

No truly objective technique exists for identifying which particular model or downscaling approach is best or most skilled in generating future climate projections for a given region and climate variable. One approach is to test each model’s ability to simulate the current climate (e.g., the last 30 years) and choose the one that provides the closest correspondence. However, there is no guarantee that this would be the “best” model for projecting the future climate. This is because various aspects of the atmospheric system, which each model handles differently, may be altered as the climate changes.

**Figure 4:**
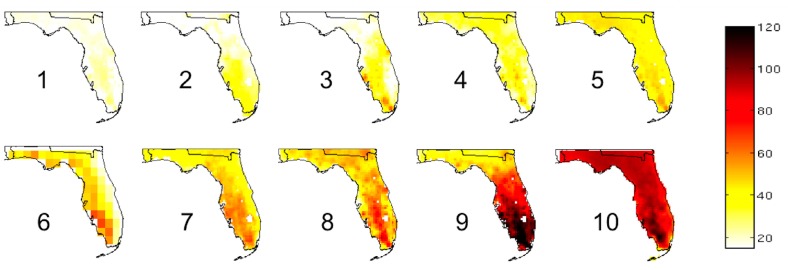
Mean change in annual number of days with a maximum temperature exceeding 35 °C (95 °F) projected between 2039–2070 and 1969–2000 for each of the 10 models used in the Florida case studies (see Table 1), arranged from least to greatest change.

Given the challenge of assessing which particular climate projection is the “best”, climatologists will often take the projections from a set of GCMs or downscaled RCMs and average them together to produce an ensemble mean value. Figure 4 shows the mean change in the annual number of days in which the temperature exceeds 35 °C (95 °F) across Florida. We compute these means by averaging the 2039–2070 projections from 10 RCM simulations and subtracting the mean model projections for the recent climate (1969–2000).

Along with a consideration of the mean change across a set of models, it is useful to identify the most and least extreme projections within a suite of climate model outputs. Using model projections that show the greatest and the least amount of change provides an envelope or range of possibilities for what may happen in the future. Figure 3b, for example, illustrates that the Florida annual mean maximum temperatures could increase by as little as 1.4 °C (2.5 °F) and as much as 3.2 °C (5.8 °F) by the period 2039–2070.

#### 2.1.1. Characteristics of Climate Projection Datasets

Numerous climate projections are available, with updates periodically released. Wooten, et al. [33] provides a detailed listing of available climate projection datasets and their attributes. The projections are constructed in a variety of ways and their data outputted across a range of spatial and temporal scales.

Each dataset has been assembled by a group of climate scientists working in a climate program funded by various government organizations, including National Oceanic Atmospheric Association (NOAA), National Center for Atmospheric Research (NCAR), and the National Science Foundation (NSF). The North American Regional Climate Change Assessment Program (NARCCAP) datasets have been used extensively in recent years and were utilized in the recent National Climate Assessment (NCA) [34].

The spatial resolution of a climate dataset defines the size of the grid boxes from which atmospheric projections are provided. These grid boxes range in size from 10 to 50 km. The Center of Climate Research (CCR) and Bias Corrected Spatial Disaggregation (BCSD) datasets are oriented around lines of longitude and latitude. The spatial domain of most models is relatively large and, for the U.S., encompasses the continental land area. The CLAREnCE10 and CCR datasets, however, are limited to the Southeast U.S. and eastern North America, respectively.

Temporal resolution defines the time increment or time step of the data, which is outputted in the form of a time series (e.g., day 1 precipitation = 0 mm, day 2 precipitation = 45 mm, etc.). The time steps of the different datasets range from hourly to monthly. Much of the research utilizing climate projections works with meteorological variables at a daily, monthly, seasonal, and annual scale. The time period(s) of each dataset span includes a recent historical period and a future period that ends either around 2070 or 2099. The historical period provides a reference or base period from which future changes can be compared.

Either a dynamical or statistical downscaling type of technique is utilized in the production of each dataset. Multiple GCMs have been used in the production of each dataset. Therefore, each dataset contains files with time series data for each GCM. Moreover, various dynamically downscaled datasets (e.g., NARCCAP) also utilize runs from a range of different RCMs. As a result, there are, for many datasets, numerous data files, each providing a projection for a given combination of GCM and RCM models. Each GCM works with one or more emissions scenarios that estimate future concentrations of pollutants and land use changes based on projected changes in economic growth, population change, and technology. The A2 and B1 emissions scenarios are typically used in climate projections. A wide variety of output variables are available in the dynamically downscaled projections, while statistically downscaled projections are largely limited to temperature and precipitation.

#### 2.1.2. Selecting Climate Projections

There are four primary considerations—diversity, number of projections, resolution, and model quality—to keep in mind when selecting climate projections for health analyses.

The first consideration is diversity. Even after applying bias correction, model projections retain errors of scientific uncertainty due to inherent imperfections. For instance, if one dataset were chosen to project disease burden, these errors would skew the disease burden projections. By averaging climatological information across multiple models, the inherent uncertainty is reduced. Alternatively, by considering solutions proposed by different models, one can delineate the full range of uncertainty in the forcing (e.g., climate) and response (e.g., health outcome). Ultimately, a diverse set of models will be less likely to suggest a systematic bias (e.g., an underestimate or overestimate of a measure of interest). The more diversity there is in the modeling suite, the more likely it is that individual model errors would—to a degree—cancel one another out when averaged [35], and that an exhaustive range of uncertainty would be obtained. Maximum model diversity is achieved by selecting non-repeating GCMs downscaled by non-repeating approaches (e.g., different RCMs, statistical downscaling techniques).

Second is the number of projections. In addition to having a diverse set of projections, having a number of them provides additional opportunity for individual errors to cancel one another out. In striving to assemble a large number of projections, however, caution should be exercised to avoid using closely related sources, such as two projections based on the same model, since this would skew the outcome towards that model [36].

Spatial resolution determines the scale of analysis. Where highly spatially resolved data are available, such projections are better able to discern local and regional climatological details associated with smaller scale processes (e.g., sea breeze influences on coastal temperature and precipitation patterns) operating in the atmosphere. Unstable models that produce high-resolution projections may not be the most appropriate to use in lieu of coarser, more stable projections.

Lastly, are considerations around model quality. It is impossible to determine a “best” model for projections despite some models’ better performance at representing past climate. A review of prior studies [36] concludes that, for the purpose of assembling a representative set of projections, the exclusion of certain models in preference to others based on past performance is not warranted. However, there is value to selecting models that may have strengths in modeling regional historical phenomena. For a region strongly influenced by El Niño, for instance, model selection may be guided by the model’s ability to produce close to the observed number and strength of El Niño events in a historical simulation.

#### 2.1.3. Determining the Magnitude of Climate Changes

In order to estimate the magnitude of climate change, the differences in the recent climate are often subtracted from the projected climate for a future time period. Table 2 provides an example.

### 2.2. Using Climate-Health Research to Estimate Present Day Health Burden

A close look at historical and present day relationships with health and climate is requisite before estimating any future health burdens. Developing causal pathways is a useful tool for identifying variables that may confound or modify relationships between an exposure of interest and a particular health outcome. Variables, including the exposure and the outcome, can be revisited and modified to reflect changes in future scenarios during sensitivity analyses for projection estimates.

Historical analyses are used to derive baseline—or present day—estimates of the relationship between a particular exposure and outcome of interest (e.g., extreme heat and hospitalizations, flooding and gastrointestinal illness). Time series (e.g., Poisson regression) [37,38,39], case-crossover [40,41], and case-only analyses [42] are epidemiological study designs typically used to extract these exposure-response functions. Generally speaking, effect estimates derived at coarser geographic scales, such as state-scale, are more stable than those at finer levels. Yet, fine scale estimates are desired because of their utility in determining spatial patterns of disease burden that are lost when aggregated to large geographic extents. Thus, there are tradeoffs between estimates at varying spatial scales. County or sub-county level estimates provide the most information relevant for developing public health and policy recommendations. Exposure data in the U.S. (e.g., daily temperature and precipitation measures) are available for most urban areas from the NOAA National Centers for Environmental Information (NCEI) [43]. Finer scale exposure data, in many cases, can be found through partnerships with local universities and private monitoring networks.

In instances where these exposure-response relationships cannot be made for a particular locale, such as where health or exposure data may not be readily available, it is appropriate to use literature-derived disease burden estimates and exposure response functions.

### 2.3. Projecting Disease Burden Using Climate Projections

#### 2.3.1. Apply Relationships Estimating Present Day Health Burden to Climate Projections

In recent years, public health researchers and practitioners have contributed substantial methodological advances for estimating future health burden due to climate change [44,45,46,47,48]. As outlined by the CDC’s guidance document for projecting disease burden, there are myriad approaches for applying present day health burdens to climate projections for future health burden estimates. The delta method [49] is a straightforward calculation wherein a previously determined exposure-response function (e.g., baseline relative risk of heat-related morbidity) is compared to a change in exposure (e.g., a 2 °C increase in temperature). This approach removes model-specific biases for both historical and projected disease burden estimates [5]. The National Research Council’s “damage function approach” is a mathematical equation that produces an estimated change in health effect by incorporating measures for baseline disease incidence, an estimated change in exposure, an estimated population exposed, and an estimated relative risk associated with the exposure and outcome of interest [50]. Similarly, the use of Environmental Protection Agency’s BenMAP tool has been combined with temperature projections to estimate the heat-related mortality by Voorhees et al. [51]. One can estimate an attributable number (AN), or the number of cases attributed to an exposure (e.g., heat-related deaths attributed to extreme heat exposure) using an attributable fraction (AF) approach [47]. The AF estimates of the proportion of cases that can be attributed to an exposure and is appealing for communicating how an intervention could lower a particular disease burden.

#### 2.3.2. Matching Scales: Climate Projections and Retrospective Climate-Health Research

Like most epidemiological investigations, in order to estimate how health impacts change in response to a unit change in exposure, there must be comparable resolution between the exposure and outcome. In the case of health burdens related to climate change, the temporal and spatial scale of the climate projection data must match that of the health data. Health data are typically available at daily or monthly resolution, with spatial scales varying from very fine (e.g., geocoded addresses of residence) to coarse (e.g., county-level aggregates). Keeping in mind that climate projection data are outputted in the form of hourly, daily, or monthly time series across grid boxes ranging between 10 and 50 km width across the earth’s surface, discordance between the climate projection data and the health outcome of interest may determine the scale at which projected health burdens can be estimated. The atmospheric variables of interest are first temporally aggregated to the time unit used in the retrospective climate-health study. This provides a time series of a meteorological variable (e.g., daily maximum temperature) that matches the time series of the health data (e.g., the daily number of heat-related ED visits). Relevant atmospheric variables for one or more grid boxes are then interpolated to the spatial units of regions defined in the retrospective climate-health study. Examples of these regions include ZIP codes, counties, regions, or an entire state.

#### 2.3.3. Make Adjustments for Projected Changes in Population Demographics

Future at-risk populations can be difficult to identify and quantify. Some In the U.S., some states have demographers that calculate state-level and, in some cases, county-level projections of populations out to mid- and next-century. Finer scale population estimates can be acquired but have temporal limitations beyond the 21st century [52]. Most projected climate-related disease burden estimates assume constant populations. While this makes the modeling somewhat less complicated, it may grossly misrepresent the likely impact on human health, particularly due to the expectation that increasingly aging populations will remain vulnerable to climate-related health impacts. In addition, considerations for how populations will adapt in the mid- and next-century are oftentimes not included in such analyses. The use of shared socioeconomic pathways (SSPs) are an increasingly touted approach for incorporating various socioeconomic scenarios that could influence human health in the next 50 or 100 years [53]. Qualitative adjustments to disease burden projections could reflect characteristics in SSP scenarios.

Total uncertainty in projected disease burden is a product of uncertainty in climate models and uncertainty in the dose-response relationship from epidemiological models.

Total uncertainty = climate modeling uncertainty × health research uncertainty

While it is nearly impossible to account for all uncertainty in models, uncertainty in disease burden projection estimates is most often addressed through sensitivity analyses. In analyses by Peng et al. [46], the authors note that the choice in climate model was the largest source of variation when calculating Chicago’s future disease burden (i.e., heat-related mortality) estimates. Uncertainty in climate models are inherent to the models themselves. As previously mentioned, using ensemble means is an acceptable approach for reducing uncertainty around exposure—or climate—variables. Similarly, ranges for the dose-response relationship and population exposure, both of which are important components to consider when projecting disease burden, can be incorporated into disease burden projections.

## 3. Florida BRACE Program Disease Burden Projections

**Figure 5:**
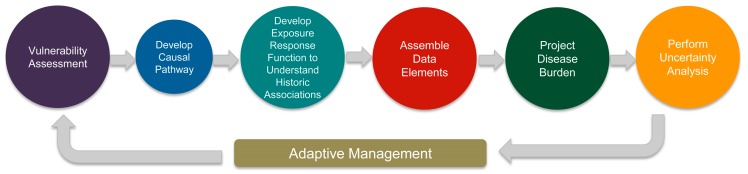
The process used by the Florida BRACE Program for developing disease burden projections. Adapted from the Centers for Disease Control and Prevention (CDC), 2016.

The CDC’s guidance document outlines four steps necessary for conducting a thorough disease burden projection exercise. The Florida BRACE Program modified this process (Figure 5), starting with a prospective vulnerability assessment using three GCM scenarios, rather than developing formal causal pathways. The assembling of the data elements focused on both temporal and geographic scale. The same regions were used for downscaled RCM data that were used for disease burden projections. When developing a suite of projections, agency programmatic priorities, strength of associations, and a mix of health outcomes (i.e., chronic, infectious, and injury) were considered. The final step of performing uncertainty analysis has yet to be completed.

### 3.1. Florida Case Study Climate Model Considerations

The Florida BRACE Program developed three case studies that use Florida-specific data to calculate baseline disease burden estimates and exposure response functions pertinent to Florida populations. The case studies provide three examples focused on: (1) the standardized precipitation index, which is a proxy for drought, and emergency department (ED) visits for asthma; (2) temperature and heat-related illness; and (3) tropical cyclones and all-cause injury. Each analysis utilizes topic-relevant health and exposure measurement data.

The Florida BRACE Program had initially chosen only four RCMs to use for disease burden projections, based on staff and resource constraints. The original models included two dynamically and two statistically downscaled models based on the A2 scenario that were thought to handle El Niño at least moderately well. After further consultation with climate scientists, it was decided that a greater number of RCMs were required to more accurately project future climate-related disease burden. Therefore, one additional dynamically downscaled model and five additional statistically downscaled models were added, for a total of 10 RCMs in the Florida BRACE model ensemble. Each projection is based on the A2 scenario but a different GCM (Table 1) to increase model diversity.

Downscaled climate projection data were obtained for all model grid boxes encompassing the state. The temporal structure of the projection data were consistent for both temperature and precipitation data. The meteorological variables were compiled as a time series (e.g., hourly and daily temperature and precipitation totals). The data were retrieved across the time intervals that corresponded to the period in which disease burden was projected: 2040–2069 and 2070–2099. Initially, two future time periods were selected; however, due to planning and resource constraints, the current work has focused only on mid-century projections.

**Figure 6:**
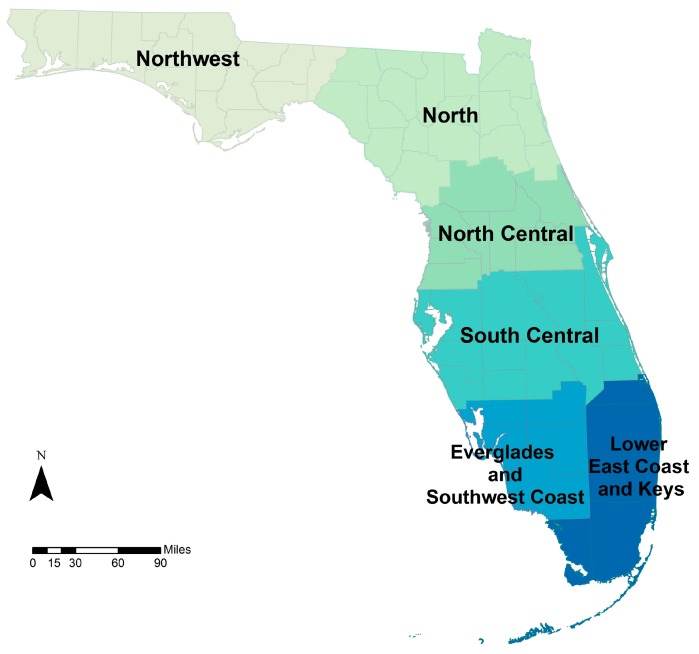
Monthly standardized precipitation index (SPI) values were estimated across six National Centers for Environmental Information (NCEI) climate regions.

Differences in spatial resolution of the climate exposure across the three case studies required additional data processing. In the drought-asthma study, it was determined that the six NCEI regions in the state (Figure 6; Lower East Coast and Keys regions were combined for projections) were the most appropriate spatial aggregation for regional precipitation patterns. Hourly to daily time series of gridded precipitation totals were obtained from the downscaled datasets and were transformed into a monthly time series of one-month standardized precipitation index (SPI) for each of the six NCEI regions. Later, rates of asthma-related ED visits were estimated across these six regions.

**Figure 7:**
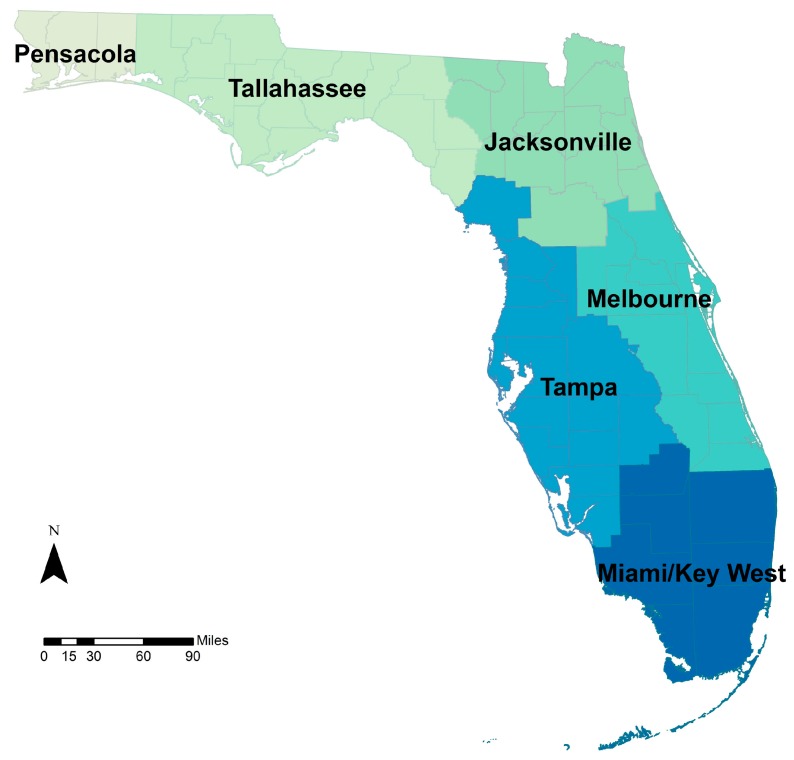
Daily maximum temperatures were determined across six National Weather Service (NWS) regions.

The heat-related illness study, was also executed for six similar, but different regions. The National Weather Service (NWS) regions (Figure 7) were determined to have similar daily maximum temperatures. Daily maximum temperatures in the downscaled climate datasets where, therefore, spatially aggregated to the NWS regions for use in the disease burden projections.

The injury and tropical cyclone study utilized a qualitative approach as no downscaled climate projection data were used. Because of the inter-annual variability and the complexity of factors associated with tropical cyclone formation, there is wide variation in tropical cyclone projections for the 21st century. The general consensus, however, is a tendency toward decreasing frequency, increasing intensity, and increasing precipitation. Therefore, disease burden projections were calculated using combined tropical cyclone impacts and hurricane only impacts using county-level data.

### 3.2. Estimating Present Day Health Burden in Florida

#### 3.2.1. Drought and Asthma Present Day Health Burden

Individual-level counts of ED visits for primary and secondary diagnoses of asthma (ICD-9-CM code 493) were aggregated to corresponding climate regions by month. Annual crude and direct age-adjusted visit rates were calculated for the study period (2005–2012) across six NCEI climate regions of the state (Figure 6). Hourly to daily time series of gridded precipitation totals from the downscaled datasets were temporally and spatially aggregated and transformed into a monthly time series of one-month SPI for each of the six NCEI regions. These constitute the baseline disease burden. A Poisson regression time series analysis for monthly rates of asthma ED visits and monthly measurements of temperature and the one-month SPI provided present day effect estimates for Florida residents. SPI provides a standardized measure of precipitation that is useful for identifying droughts and unusually wet periods. These analyses were completed for other disease outcomes of interest (e.g., respiratory, food- and waterborne disease) and are available on the Florida Department of Health website [54].

#### 3.2.2. Extreme Heat Illness Present Day Health Burden

Individual-level counts of ED visits for heat-related illness (HRI) (ICD-9-CM codes 992, E900) were aggregated to postal ZIP code level by day of ED visit. Annual crude and direct age-adjusted visit rates were calculated for the study period (2005–2012). A Poisson regression time series analysis for daily rates of HRI linked with the nearest weather station daily maximum temperature provided effect estimates at the climate region level (Figure 7) for Florida residents. Estimates at the state level were also calculated using a meta-analysis technique to aggregate the regional estimates [55]. Daily maximum temperatures in the downscaled datasets were, therefore, spatially aggregated to these regions for disease burden projections. These analyses were also completed for other health outcomes of interest, such as cardiovascular and respiratory disease (results not shown).

#### 3.2.3. Tropical Cyclone and All-Cause Injury Present Day Health Burden

Individual-level counts of ED visits for all-cause injury were obtained and aggregated to the county level by day of visit. A hybrid matched cohort analysis was conducted to produce effect estimates [56]. Exposure periods (i.e., counties experiencing at least tropical storm-force winds) were matched to a pre- and post-hurricane season control period (i.e., no tropical storm-force winds) by county and day. These analyses were also completed for other health outcomes of interest, such as carbon monoxide poisoning and food- and waterborne diseases.

### 3.3. Estimating Future Heat Related Illness Health Burden in Florida

We present results from the temperature and HRI disease burden projection exercises from the BRACE project. The tropical cyclone-related disease burden projections have been presented elsewhere (96th American Meteorological Society Annual Meeting), and the drought (SPI) and asthma projections are ongoing.

**Figure 8:**
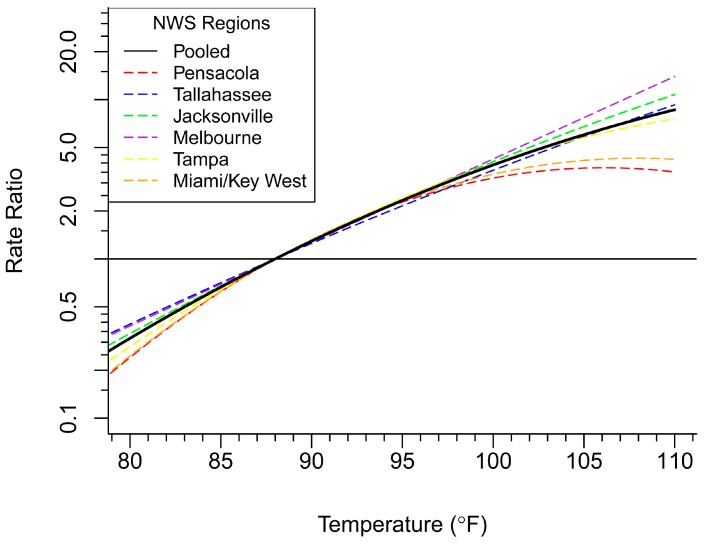
Estimated rate ratios of heat-related illness for temperatures, relative to the 88 °F threshold, for six Florida National Weather Service (NWS) regions and statewide.

Projected heat illness is a climate-related concern that many health departments are faced with preparing for and adapting to. In this case study, we estimated projected heat-related illness disease burden using an AF approach. AFs were calculated for each value of maximum temperature (T_max_) above a reference range of 88 °F (average T_max_ for Florida during historical period 2005–2012). Rate ratios were calculated for each temperature above the 88 °F threshold for the six NWS regions and pooled for a statewide estimate (Figure 8).

Attributable numbers (AN) of HRI cases per year were calculated based on the formula below:

$$AN=\;\sum^{\text{​}}\left[AF\left(T_{i}\right)\;\times MDC\;\times ND\left(T_{i}\right)\right]$$

where MDC = mean daily count of HRI and ND(T_i_) = number of days with a value of the at or above the 88 °F threshold [47]. Table 3 presents disease burden projection results by NWS region for the average number of HRI cases occurring above the threshold of 88 °F per year for the period of interest (2040–2069), averaged across all 10 GCMs. These estimates represent the average number of excess HRI cases per year due to projected change in the maximum daily temperature, across all regions. Some regions will be affected by climate change more than others, and thus, will have a larger future burden of disease attributable to those changes.

**Figure 9:**
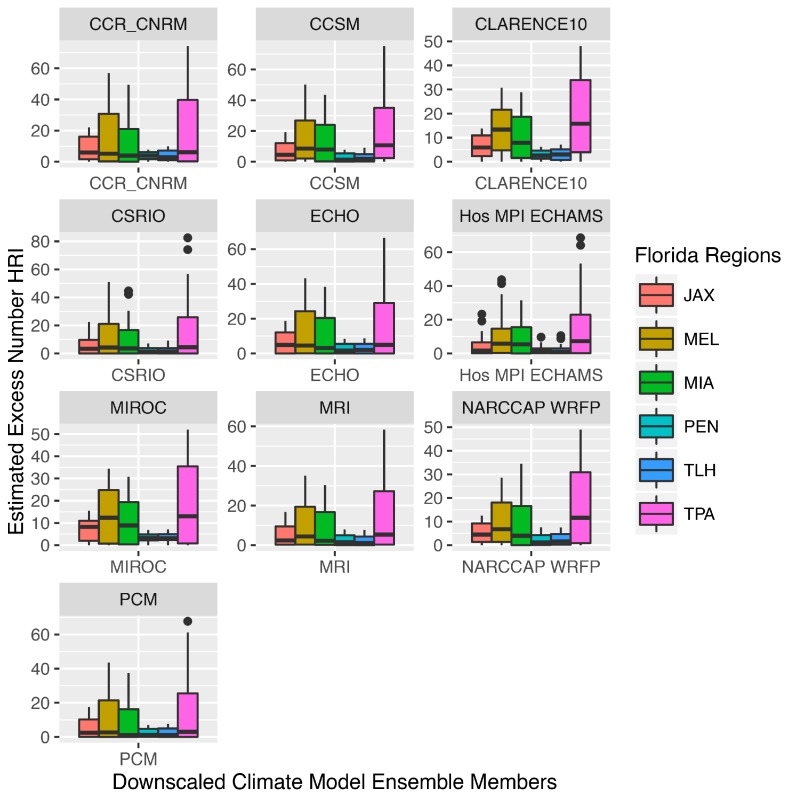
Estimated excess number of heat-related illness by downscaled Florida regional climate model for six Florida regions.

Variation in model estimates across GCMs are presented in Figure 9. Individual ensemble members, in some cases (e.g., CLARENCE 10, NARCCAP), report higher numbers of projected HRI across most regions. By averaging the 10 models, the estimates account for the outliers and differences in model means.

## 4. Discussion

Studying the associations between climate and health outcomes is a relatively new concept, and projecting future disease of climate-related outcomes from within a state or local health department is novel. While public health has significantly expanded its scope in the last century from a focus on overall environmental health, to acute and communicable diseases, to chronic conditions and risk behaviors, the majority of funding and capacity within agencies tends to be focused on primary disease surveillance and prevention efforts. These are fundamental public health functions that can potentially supply data for climate and health programs and analyses. However, it also means that too often staff and agency leadership do not have a complete understanding of, an appreciation for, or specific training and technical skills related to the study of climate and health [57].

To successfully incorporate short and long-range climate projections into public health planning and response activities, a public health agency needs an interdisciplinary team with highly technical skills. The Florida BRACE collaborative included staff with traditional epidemiological training, staff with backgrounds in other disciplines (i.e., health education, environmental science, urban planning, demography, sustainability), contractors, and partners with highly technical geographic, climatological, and meteorological skills. The value of a strong partnership with state or regional climatologists and climate modelers cannot be overstated. While nearly all of the environmental and climate data needed to successfully complete climate-related disease burden projections are freely available, navigating complex web and FTP sites, understanding the breadth of information available, and manipulating the data into a format that is compatible with public health data, requires expertise that is usually housed outside a public health agency.

Disease burden projections can strengthen public health preparedness and response plans around climate-sensitive health outcomes. The projections can be used in conjunction with other information about diseases that are common among a locale’s population. For instance, one way the HRI projections could be used is in the development county-specific near-future heat adaptation or response plan. Counties that have rising rates of renal disease, a disease which is associated with heat-related illness [58], may want to know the magnitude in which HRI is expected to occur so that targeted adaptation plans can develop strategies to focus on those at-risk populations in the near and extended future.

There are inherent limitations that constrain this work, including expertise, data availability, funding, and time. Managing the effort requires sensitivity to resource constraints, partner expectations, and agency priorities. Substantial time and effort can be saved by utilizing existing research, such as downscaled regional climate projections, and collaborating with local partners on topically similar projects. Most disease burden projections focus on temperature and heat-related illness. Other climate-related outcomes such as vectorborne disease (e.g., Lyme disease or West Nile virus) are garnering attention from public health practitioners. More research is needed to identify the best methods for projecting vectorborne diseases, at varying temporal and spatial scales, and how to interpret and use those projections in public health practice. Partnering with subject matter experts who have projected disease burden, for instance those with experience projecting extreme heat disease burden, could be a useful approach for health outcomes that have not yet been projected.

Resource limitations, however, should not dissuade one away from disease burden projection exercises. A powerful communication tool is being able to describe how the present day disease burden relates to climatic conditions, and further expounding on how those conditions will change in the future. Qualitative estimates can support climate adaptation planning through phased efforts that follow an adaptive management strategy, whereby modifications can be made as more information and resources become available. Ultimately, the necessary elements for success include an enthusiastic staff with diverse skillsets, strong partnerships, and a scope of work that reflects the resources and support dedicated to developing disease burden projections.

## 5. Conclusions

A basic understanding of climate change, GCMs, downscaling, and the uncertainty inherent in climate modeling is crucial for public health practitioners aiming to project climate-related disease burdens. It is unrealistic for public health practitioners to have a full working knowledge on technical processes related to climate change and GCMs, which is why it is imperative to establish productive, respectful, and mutually beneficial working relationships with experts from other fields.

This article is the first of its kind, dedicated to providing public health practitioners with a primer on the key points for understanding and decision-making from the climate science perspective. We intentionally include technical nuances on both sides of climate and health analyses to demonstrate the need for developing collaborations across expertise. The case studies illustrate three approaches for utilizing climate projection data to estimate projected disease burden. By using ten climate models for projecting HRI in the mid-century, we were able to illustrate how climate model parameters differ across models, yielding a range of disease burden estimates. Although climate models have uncertainty in their projections, we provide various ways to reduce uncertainty as well as how to interpret climate model outputs so that they can be used in public health. Results from disease burden projection exercises can play critical roles in the development of adaptation and response strategies for public health practitioners and agencies concerned about climate-related exposures. There are opportunities to expand this area of research and practice into additional areas of climate and health outcomes.

## Figures and Tables

**Table 1:** Downscaled Coupled Model Intercomparison Project (CMIP)3-A2 scenario datasets used by Florida Building Resilience Against Climate Effects (BRACE).

Dataset	Time Period(s) Available	GCM	Downscaling Approach	Spatial Resolution
CCR ^1^	1961–2000, 2046–2065, 2081–2100	CNRM	Statistical	11 km
CLAREnCE10 ^2^	1969–2000, 2039–2070	GFDL	Dynamical (RCM: RSM)	10 km
Hostetler ^1^	1960–2099	MPI-ECHAM5	Dynamical (RCM: RegCM3)	15 km
NARCCAP ^3^	1969–2000, 2039–2070	CGCM3	Dynamical (RCM: WRFG)	50 km
SERAP ^1^	1960–2099	CCSM, CSIRO, ECHO, MIROC, MRI, PCM	Statistical	12 km

^1^ Downloaded from [30]; ^2^ Downloaded from the Florida Climate Institute [31]; ^3^ Downloaded from North American Regional Climate Change Assessment Program [32].

**Table 2:** Example of corresponding magnitude of change in base and projection time periods for days exceeding T_max_ of 35 °C (95 °F).

Time Period	Number of Days in Which the Daily Maximum Temperature Equals or Exceeds 35 °C (95 °F)
Future Projection: 2070–2099	75
Base Climate: 1990–2009	50
Change	25 (50% increase)

**Table 3:** Projected additional heat-related illness cases per year, by NWS region averaged across 10 global circulation models.

Region	Projected Additional Heat-Related Illness Cases per Year (2040–2069)	
	Average	Range
Pensacola	64	44–102
Tallahassee	67	45–102
Jacksonville	145	112–208
Melbourne	296	220–404
Tampa	422	347–529
Miami/Key West	244	180–341
